# Evaluation of Nutritional and Antioxidant Status of *Lepidium latifolium* Linn.: A Novel *Phytofood* from Ladakh

**DOI:** 10.1371/journal.pone.0069112

**Published:** 2013-08-02

**Authors:** Tarandeep Kaur, Khadim Hussain, Sushma Koul, Ram Vishwakarma, Dhiraj Vyas

**Affiliations:** 1 Academy of Scientific and Innovative Research, Indian Institute of Integrative Medicine (CSIR), Canal Road, Jammu, India; 2 Biodiversity and Applied Botany Division, Indian Institute of Integrative Medicine (CSIR), Canal Road, Jammu, India; 3 Medicinal Chemistry Division, Indian Institute of Integrative Medicine (CSIR), Canal Road, Jammu, India; University of Westminster United Kingdom

## Abstract

*Lepidium latifolium* Linn. (perennial pepperweed) is one of the preferred phytofoods among cold arid region of Ladakh, India and its leaves contribute significantly to people's diet. This study was conducted to determine its nutritive value and antioxidant activity. Plant samples from three different locations were selected in the present study. Results showed that this plant is an excellent source of glucosinolates, notably sinigrin that is present in very high amount (∼70–90%). Its value ranged from 149 to 199 µg per g fresh weight. Fatty acid composition analysis showed that its leaves were abundant in unsaturated fatty acids, specifically linolenic acid (18∶3) whose percentage is about 50%. Higher glucose and crude protein along with higher nitrogen to sulfur ratio, supplements the nutritive value of this plant. Based on total phenol, flavanoids, free radical scavenging activity and DNA protective activity showed that this ecotype of perennial pepperweed contains high antioxidant properties. The percentage inhibition for O_2_^−^ scavenging activity ranged from 41.3% to 83.9%. Higher content of phenols (26.89 to 50.51 mg gallic acid equivalents per g dry weight) and flavanoids (38.66 to 76.00 mg quercetin equivalents per g dry weight) in leaves could be responsible for the free radical scavenging activity of this plant. Depending upon the location of the plants, variations were observed in different activities. Based on the systematic evaluation in this study, preparations of *Lepidium latifolium* from Ladakh can be promoted as substitute to dietary requirements.

## Introduction

Living organisms have to relate themselves to the environment for their sustenance. Environmental factors and metabolic processes in the body cause molecules or atoms to form free radicals that have increased chemical reactivity. Today's oxidative environment presents a range of free radicals including superoxide, hydroxyl radical, nitric oxide and peroxynitrite, for living organism to deal with. There has been a plethora of concrete scientific evidence that support the important role of free radicals in development of some diseases like cancer [1], neurodegenerative diseases [2] and inflammatory diseases [3]. Antioxidants have therefore gained importance due to their ability to neutralize free radicals or their effects. They may act as free radical scavengers, reducing agents, chelating agents for transition metals, quenchers of singlet oxygen molecules and/or activators of antioxidative defense enzyme systems to suppress the radical damages in biological systems.

Humans make many antioxidants themselves (examples being superoxide dismutases, catalases, reduced glutathione, and peroxiredoxins and obtain some other actual (e.g., vitamin E, ascorbate) or putative (e.g., flavonoids, carotenoids) antioxidants from the diet [4]. Therefore, it is important to take a balanced antioxidant rich diet for defending our body against free radical oxidative damage. Although, dietary fruits and vegetables have sufficient antioxidants under normal situations [5], additional antioxidant supplements are required under diseased and physiologically challenged situations. In fact, it has now been emphasized that supplementing antioxidants might sometimes cause harm rather than good [4], [6]. Hence, there is increasing interest in naturally occurring antioxidants for use in food and pharmaceutical industry to replace the synthetic antioxidants. In this regard, *phytofoods* play an important role because of their traditional uses over centuries.

*Lepidium latifolium* Linn. is one such important plant having ethnic value as *phytofood*. It is also known as perennial pepperweed and belongs to family Brassicaceae. This plant is native to southern Europe and Asia [7], [8]. It grows between 30 cm and 2 meters and has woody stems, waxy leaves and small white ﬂowers arranged in clusters. In Ladakh (31°44′57′–32° 59′57′ N latitude and 76° 46′29′–78° 41′ E longitude), it grows naturally at altitudes ranging from 2500 m to 4500 m asl. This Himalayan cold arid region represents unique ecosystem characterized by severe environmental conditions such as sub zero temperatures, low annual precipitation (80–300 mm mostly in form of snow), intense radiation load, varying moisture levels and low partial pressure of gases [9]. These severe conditions would load the plants with antioxidants and novel mechanisms for its survival. In fact, there have been recent reports that understand anti-stress mechanism in this cold tolerant plant [10], [11].

Leaves of this plant are mainly consumed as garnish and *shangso chonma*, its traditional Ladakhi dish, is considered best among the known local dishes of wild edible plants [12]. The vegetative part of *Lepidium latifolium* is present for about 7–8 months, unlike other plants of Ladakh area that show growth for about 3–4 months. It therefore forms an important part of traditional diet under harsh conditions. It is hypothesize that this ecotype of the plant would be a rich source of glucosinolates. Although, produced by the plant for ecological roles, glucosinolates have profound nutritional and anticancer effects [13], [14]. Perennial pepperweed has already been reported to have therapeutic properties as diuretic [15] and anti-hypertensive [16]. Although, the plant is used as *phytofood* by people in that area from times immemorial, there is no study in literature that focuses on its antioxidant and nutritional effects. There are some reports on preparations and their uses in food and beverages of this plant. The present study is therefore an attempt to present a systematic nutritional and antioxidant study of this important *phytofood*.

## Materials and Methods

All chemicals and standards used in the present study have been procured from Ms/- Sigma-Aldrich Co., St. Louis and Ms/- Sisco Research Laboratories Pvt. Ltd., Mumbai, unless otherwise stated.

### Plant Material

**Figure 1:**
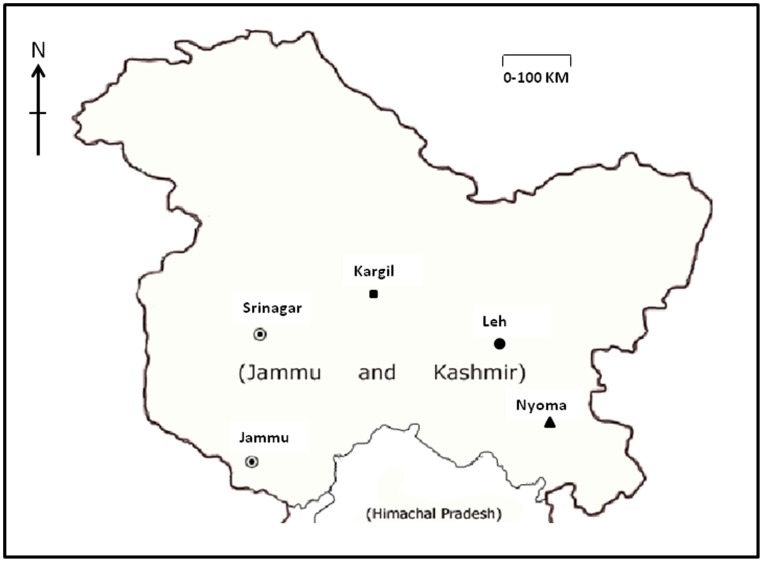
Locations from where the plants of Lepidium latifolium were collected from Ladakh region of Jammu and Kashmir State, India; Kargil (▪), Leh (•), Nyoma (▴). The figure is for illustrative purposes only.

Naturally occurring populations of *Lepidium latifolium* were identified and mature plants at the initial flowering stage were selected for various assays. Plant were selected from Kargil (34.54 N, 76.13 E), Leh (34.15 N, 77.57 E) and Nyoma (33.20 N, 78.64 E), representing three different microclimatic zones within Ladakh region (Fig. 1). Plant material was collected on a bright sunny day at 10.00 hrs and immediately stored in liquid nitrogen till further analysis. Prior permission of Forest Department, Jammu and Kashmir State, India was obtained before conducting the study.

### Preparation of crude methanol extract

500 mg of leaves and roots of plants from all three locations were crushed using liquid nitrogen. Three different leaf positions viz., top, middle and bottom, were used for all the studies. Extracts were prepared using fine powder with 10 mL of 80% methanol at 40°C by continuous stirring for 8 h. This was repeated thrice; extracts were pooled, filtered, and dried using rotary vacuum concentrator and lyophilizer [17]. The lyophilized extracts were finally dissolved in methanol and diluted for various working concentrations. Extracts in equal concentration (500 µg) was used for each sample in all the antioxidant assays.

### Glucosinolate analysis

Glucosinolates were extracted from one gram of tissue using hot methanol (70%) method as described previously with some modifications [18]. Glucosinolates were analysed using HPLC as desulpho-glucosinolates after treating them overnight with sulfatase. Sinigrin was added as an internal standard and used for quantification of various desulfo products. The HPLC was done on Agilent 1260 Infinity Quaternary LC System equipped with a ZORBAX Eclipse Plus C18 column (250 mm ×4.6 mm, 5 µm) using a flow rate of 1 ml min–1. The desulpho-glucosinolates were eluted by the following gradient: H_2_O (2 min), a linear gradient of 0–60% methanol (48 min), a linear gradient of 60–100% methanol (3 min), and 100% methanol (3 min). Individual glucosinolates were identified by comparison to and spiking with authentic standards, and comparison to elution profiles of previously published chromatograms. Quantification was done relative to the internal standard, using published relative response factors at 229 nm according to the standard ISO 9167-1 method.

Desulfo moieties of known standards were also prepared using similar procedure and their elution time was recorded under above chromatographic conditions. Authentic standards of glucotropaeolin, gluconapin, glucoraphanin, glucocheirolin, sinigrin, glucoiberin, glucobrassicin and phenylethyl glucosinolates were all procured from Ms/- ChromaDex®, Irvine, CA, and used for above analysis.

### Fatty acid methyl esters (FAME's)

The fatty acid composition of the extracts was analyzed as described by Garcés and Mancha [19]. 200 mg of plant material was crushed in liquid nitrogen and heated with a reagent containing methanol:heptane:benzene:2,2-dimethoxypropane:H_2_SO_4_ (37∶36∶20∶5∶2, v/v). Simultaneous digestion and lipid transmethylation took place in a single phase at 80°C. After they were cooled at the room temperature, the upper phase containing the FAMEs was taken for GC-MS analysis. Analysis was performed on a GC-MS 4000 system (Varian, USA) equipped with Supelco capillary column (15m ×0.32 mm ×0.25 µ). The initial temperature of 150°C for 2 min was raised to final temperature of 280°C at a rate of 3°C min^−1^. Carrier gas was helium at a ﬂow rate of 1 ml min^−1^. During the analysis, the temperature of injector was maintained at 280°C. The MS scan parameters included electron impact ionization voltage of 70 eV, a mass range of 40–500 m/z. The identification of FAME's was based on comparison of their mass spectra with those stored in NIST05 library or with mass spectra from literature.

Calculated oxidizability value (Cox) was calculated based on the percentage of unsaturated C18 fatty acids, applying the formula proposed by Fatemi and Hammond [20].

Cox  =  [1(18∶1%) +10.3(18∶2%) +21.6(18∶3%)]/100

### Glucose and protein analysis

Total glucose was estimated using glucose assay kit (GAGO-20) from Ms/- Sigma-Aldrich Co., St. Louis, which was specifically meant for food material. Briefly, H_2_O_2_ is generated by the action of glucose oxidase on glucose that converts colourless reduced dianisidine to coloured oxidised form. Intensity of the colour is stabilized using H_2_SO_4_ and its intensity was measured at 540 nm. A standard plot of D-glucose was prepared ranging from 0.1–1 ng (R2 = 0.9901) and sugars were expressed as ng g^−1^ fresh weight.

Total crude protein was estimated using Bradford reagent. Total protein was precipitated using 20% TCA solution and after centrifugation dissolved in 1N NaOH. Total protein was compared using BSA standard curve (0.5–5 µg, R^2^ = 0.969) and was expressed as mg g^−1^ fresh weight.

### Elemental analysis

Elemental analysis was performed using Vario EL Ver III CHNS analyser from Elemental Analysensysteme, GmbH, Germany using thermal conductivity detector strictly as per manufacturer's instructions. Elemental carbon, hydrogen, nitrogen and sulfur were estimated from 5 mg of lyophilized leaves and roots.

### Total phenolic content

Total phenolic content was determined as described by Prior et al. [21]. Brieﬂy, 500 µg of extract in 100 µL of methanol was mixed with 100 µL of 1 N Folin–Ciocalteu reagent. Following incubation for 5 min, 200 µL of 20% Na_2_CO_3_ was added. Absorbance at 730 nm was measured in plate reader after 10 min and the concentration of phenolic compounds was calculated using standard curve of gallic acid (500–5000 ng; R^2^ = 0.967). The results were expressed as mg gallic acid equivalent (mg GAE) g^−1^ dry weight of plant material.

### Total flavanoids

For flavonoid content, extract in 500 µL of distilled water was mixed with 30 μL of a 5% NaNO_2_ solution and incubated for 5 min. 300 μL of 10% AlCl_3_.H_2_O solution was added followed by 200 μL of 1 M NaOH and 200 μL of distilled water after 6 min. Absorbance was read at 510 nm and total ﬂavonoids were calculated using quercetin as standard (10–100 μg; R2 = 0.999). The results were expressed as mg quercitin equivalent (mg GAE) g^−1^ dry weight of plant material.

### O_2_^−^ scavenging activity

Super oxide anion (O_2_^−^) scavenging activity was performed based on NBT reduction assay essentially as described earlier [22]. The reaction (300 μl) was performed in phosphate buffer (50 mM; pH 7.8) containing 500 ug of extract, 1.5 μM riboﬂavin, 50 μM NBT, 10 mM DL-methionine, and 0.025% v/v Triton X-100. Reaction was initiated by illuminating the reaction mixture and absorbance of formazan was recorded at 560 nm. Percentage scavenging activity was described as inverse of the produced formazan.

### Other antioxidant activities

1,1-diphenyl-2-picrylhydrazyl (DPPH) radical scavenging assay, hydroxyl radical (OH^·^) scavenging activity, reducing power assay and chelation power on ferrous (Fe^2+^) ions on all extracts was performed essentially as described earlier [17], with a few modifications for plate reader analysis.

### DNA protective assay

Hydroxyl radical induced DNA breakage in plasmid pUC 18 was done as described earlier [17]. Brieﬂy, 5 μL of the above mentioned extract was mixed with freshly prepared Fenton's reagent (50 mM phosphate buffer, 1.6 mM FeSO_4_, 3 mM EDTA and 3 mM H_2_O_2_) and 1 uL of pUC 18 plasmid (250 ng). The final volume of the reaction mixture was brought to 15 μL with de-ionized distilled water and incubated for 30 min at 37°C. Plasmid DNA products were visualized in EtBr stained agarose gel under illumination of UV light and photographed with Gel Doc system (UVP Ltd., Cambridge, U.K.). The densitometry analysis of the gel was performed using *Alpha DigiDoc 1201* software (Alpha Innotech Corporation, CA, USA) and total integrated density values of bands were calculated.

### Statistical analysis

The data obtained in this study were expressed as the mean of triplicate determinations and standard deviation (SD). Statistical comparisons were made using one-way ANOVA followed by Tukey's HSD test. Analysis was done using IBM® SPSS® Statistics version 20.0 program and values of *p≤0.05* were considered to be significant.

## Results and Discussion

Trans-Himalayan plants have traditionally been known as valuable source of antioxidants and nutritive values. There are many reports that suggest the use of plants growing in cold arid zone of Himalayas as source of *phytofood* [9], [12], [23]. In most of these studies, the basis of useful properties has been the traditional practices followed in the Indian System of Medicine. Recently, efforts have been focussed on scientific evaluation of these plants. The ecotype of *Lepidium latifolium* from Ladakh is one such plant that is considered best among local wild edible plants of Ladakh [12]. Cold tolerance ability of this plant has recently been tapped using molecular techniques [10], [11], [24]. The present investigation is an attempt to evaluate its antioxidant and nutritional properties with an aim to utilize this information as guide and aid in its marketing efforts.

Methanolic extract was used for determination of various properties of this plant, because maximum yield was observed in this fractions (data not shown), as reported in other plants also [17], [25]. The extract yield varied from sample to sample and was found in the range of 6.1% (Nyoma roots) to 13.0% (Nyoma top leaves) as given in table 1.

**Table 1:** Relative proportion of methanolic fraction in different position of leaves and roots of *Lepidium latifolium* expressed as percentage of total amount of crude extract used for the fractionation (n = 3).

	Kargil				Leh				Nyoma			
	Leaves			Roots	Leaves			Roots	Leaves			Roots
	*Top*	*Middle*	*Bottom*		*Top*	*Middle*	*Bottom*		*Top*	*Middle*	*Bottom*	
Total Yield (mg)	55.2	41.0	42.2	46.1	39.7	33.8	38.2	55.3	65.1	32.1	49.9	30.7
% Yield	11.0	8.2	8.4	9.2	7.9	7.6	6.7	11.0	13.0	6.4	8.5	6.1

Each value is a mean of a triplicate analysis performed on different samples.

### Glucosinolate analysis

**Figure 2:**
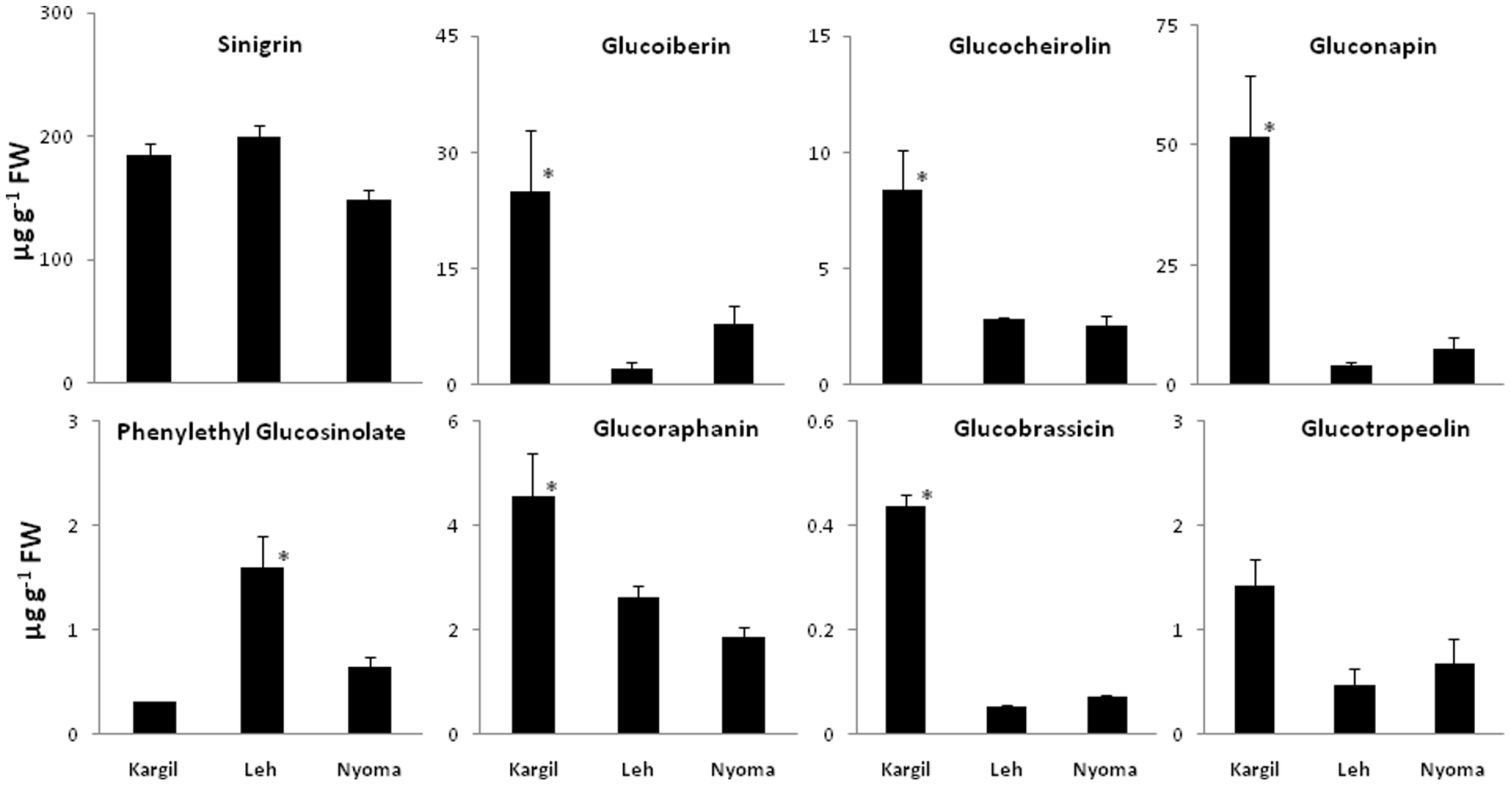
Metabolic content of eight reference glucosinolates in leaves of *Lepidium latifolium* at three different locations of Kargil, Leh and Nyoma. * represents statistically significant values at *p≤0.05*.

**Figure 3:**
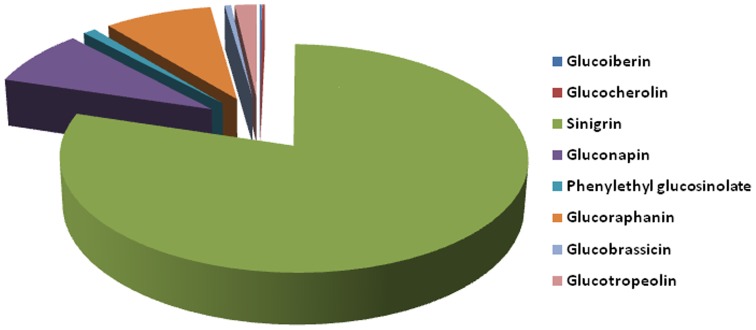
Representation of the major glucosinolates in native plant species of *Lepidium latifolium* in Ladakh. Values represent the average of all the three locations.

There is plethora of literature available to substantiate that glucosinolates and their degradation products are known source of health alleviating properties [13], [26], [27]. Belonging to a very unique region of Ladakh and being a member of family brassicaceae, *Lepidium* is expected to be rich in glucosinolates. Eight different glucosinolates representing different classes were identified in HPLC analysis of desulfo moieties of glucosinolates in the top leaves. These include glucoiberin, glucobrassicin, glucocherolin, glucoraphanin, sinigrin, gluconapin, glucotropeolin and phenylethyl glucosinolate. An allyl- glucosinolate namely sinigrin is the major glucosinolate observed in this plant (Fig. 2). Its value was found to be in the order Nyoma (149 µg g^−1^) < Kargil (185 µg g^−1^) < Leh (199 µg g^−1^) on fresh weight basis. Out of all the identified seven glucosinolates, its percentage was about 90% in Leh (Fig. 3). Earlier, it was found to be 90% in *Brassica juncea* L. integlifolia group [28]. This is found to be one of the largest sources of sinigrin among plants. Other abundant sources include wasabi, mustard and horseradish [29], [30]. Our study reports comparable or better amount of sinigrin in leaves of *Lepidium latifolium*. Other notable glucosinolates observed in the present study are glucoiberin, gluconapin and glucocherolin. Health effects of glucosinolates are notably credited to isothiocyanates, which are their hydrolysis products. They have been shown to provide protection against carcinogenesis, cardiovascular and CNS disorders, restoration of skin integrity, and protection against *Helicobacter pylori* infections [13]. The hydrolysis product of sinigrin, allyl isothiocyanate (AITC) has been particularly shown to provide antimicrobial and anticancer activities [31]. These health beneficial effects substantiate the need for looking at newer sources of glucosinolates with higher efficacy. Our investigation shows this particular ecotype of *Lepidium latifolium* from Ladakh to be an important source of glucosinolates, notably sinigrin. Natural selection from wild varieties of crucifers has always been exploited for quantitative and qualitative variations among glucosinolates [32]. When content of glucosinolates was compared among different sites, variations were observed which may be credited to the micro-climate prevalent in that area. As these are natural populations, subtle phenological changes are expected that may lead to qualitative and quantitative differences in glucosinolates content.

### Fatty acid composition

GC-MS analysis was performed on methyl esters extracted after digestion of fresh tissue and trans-methylation of lipids [19]. The fatty acid composition from this plant is summarized in table 2. It indicates that leaves of this plant are abundant source of polyunsaturated fatty acids (PUFA), having highest content of linolenic acid (18∶3). The amount decreases as we go from top to bottom position suggesting that the fresh leaves and twigs are the best source of PUFA. This goes well with the traditional practice of using these parts in local cuisine preparations and is nutritionally very favourable. Other important unsaturated fatty acids include linoleic acid (18∶2) and oleic acid (18∶1). Apart from these, roots also contain palmatolic acid (16∶1) which is present in small quantities. Meanwhile, major saturated fatty acid (SFA) is palmitic acid (16∶0) which comprised approximately 20–30% of total fatty acids (Table 2). Another major SFA present in this plant is stearic acid (18∶0) with quantities ranging from 10–30%. Small quantities of myristic acid (14∶0) and lauric acid (12∶0) were also observed in leaves, whereas, roots also contain 1–7% of pantadecanoic acid (15∶0). In general, the fatty acids follow a pattern with Nyoma plants showing highest quantities followed by Leh plants followed by plants growing in Kargil.

**Table 2:** Percentage (%) fatty acid composition of *Lepidium latifolium* from different locations of Ladakh.

	Kargil				Leh				Nyoma			
	Leaves			Roots	Leaves			Roots	Leaves			Roots
	*Top*	*Middle*	*Bottom*		*Top*	*Middle*	*Bottom*		*Top*	*Middle*	*Bottom*	
**C12:0**	1.15	0.93	2.11	1.39	1.05	1.29	0.68	1.17	0.77	1.01	1.97	1.78
**C14:0**	0.99	1.11	1.69	1.29	1.04	1.25	0.76	1.19	0.78	1.16	1.76	1.16
**C15:0**	–	–	–	1.12	–	–	–	6.54	–	–	–	7.00
**C16:0**	26.21	27.99	30.34	25.39	24.41	25.74	22.48	19.14	23.61	24.59	31.32	20.53
**C16:1n-7**	–	–	–	0.31	–	–	–	0.38	–	–	–	0.29
**C18:0**	17.50	22.85	29.49	30.38	16.40	21.77	12.70	17.55	10.73	15.88	32.95	17.85
**C18:1 n-9**	1.61	2.13	1.85	4.36	2.45	2.39	2.15	1.61	1.03	1.37	1.91	4.07
**C18:2 n-6**	5.18	6.55	4.30	5.23	5.70	5.02	6.53	3.62	6.28	3.99	2.75	3.65
**C18:3 n-3**	47.33	38.41	30.18	9.72	48.02	42.52	54.67	7.39	56.76	51.97	27.31	12.37
**∑SFA**	45.86	52.90	63.65	59.57	42.91	50.06	36.63	45.60	35.90	42.65	68.01	48.34
**∑MUFA**	1.61	2.13	1.85	4.67	2.45	2.39	2.15	1.99	1.03	1.37	1.91	4.36
**∑PUFA**	52.51	44.96	34.49	14.95	53.73	47.54	61.21	11.02	63.05	55.96	30.06	16.02
**∑PUFA/SFA**	1.14	0.84	0.54	0.25	1.25	0.94	1.67	0.24	1.75	1.31	0.44	0.33
**COX Value**	10.77	8.99	6.98	2.68	10.98	9.72	12.50	1.98	12.91	11.65	6.20	3.08

C12:0, lauric acid; C14:0, myristic acid; C15:0, pentadecanoic acid; C16:0, palmitic acid; C16:1 n-7, palmitoleic acid; C18:0, stearic acid; C18:1 n-9, oleic acid; C18:2 n-6, linoleic acid; C18:3 n-3, linolenic acid; SFA, saturated fatty acid; MUFA, monounsaturated fatty acids; PUFA, polyunsaturated fatty acids; Cox: calculated oxidizability value. Each value is a mean of a duplicate analysis performed on different samples.

Polyunsaturated fatty acids such as linoleic acid (18∶2) and linolenic acid (18∶3) are called essential fatty acids (EFA) because of their necessity in the human body. Linolenic acid is a known enhancer for transporting bioactive compounds into the skin, and it is converted to arachidonic acid which serves as a precursor for powerful hormone-like compounds [33]. According to current dietary guidance for healthy nutrition, higher PUFA/SFA ratio is associated with good health (http://www.health.gov/dietaryguidelines/). As seen in table 2, this ratio is more than 1 in top leaves with plants growing in Nyoma showing the value of 1.75. Similarly, the Cox values that act as nutritive index are significantly higher in this plant. Its value ranged from 6.2 to 12.9 in leaves at different positions and cultivation sites. PUFA/SFA ratio and Cox values in roots however, showed lower values. Cox values in top leaves of this plant are almost double than found for fruits of *Pistacia* in a recent report [34], thereby making it one of highly nutritive *phytofood*.

### Elemental analysis

When elemental analysis was performed in this plant, it was observed that the percentage of S was very high. No significant difference was found in the C and H percentage among leaves and roots, whereas, leaves contain lesser %N and %S than roots making them sink of these nutrients (Table 3). %N found in top leaves were found in the range of 2.9 to 4.1, whereas %S ranges from 1.1 to 3.1. It has been proposed that N:S ratio is a true measure of protein quality in human diets rather than their individual content [35]. For most plants, the optimum ratio considered was found 10∶1–15∶1, especially for ruminant nutrition [36]. The N:S ratio in leaves of this plant was observed between 1∶1 to 3∶1 suggesting it to be very nutritive. Humans and animals must be supplied with S amino acid methionine and the S- bearing vitamins biotin and thiamine [37].

**Table 3:** Elemental analysis in leaves and roots of *Lepidium latifolium* from three different locations of Ladakh.

	Leaves			Roots		
	Kargil	Leh	Nyoma	Kargil	Leh	Nyoma
**%C**	33.41±1.30	41.53±0.57	40.23±0.28	42.37±1.62	41.94±0.48	40.76±2.10
**%H**	5.54±0.13	6.29±0.06	5.98±0.03	6.32±0.14	6.15±0.13	6.00±0.11
**%N**	2.91±0.21	3.07±0.20	4.15±0.17	1.74±0.18	0.59±0.15	0.61±0.12
**%S**	3.12±0.15	1.10±0.17	1.44±0.12	0.40±0.02	0.44±0.17	0.20±0.04
**N:S**	0.93	2.79	2.88	4.35	1.34	3.05

%C: percentage carbon; %H: percentage hydrogen; %N: percentage nitrogen; %S: percentage sulfur; N:S: nitrogen to sulfur ratio. Each value is a mean of a duplicate analysis performed on different samples.

### Total glucose and protein

**Figure 4:**
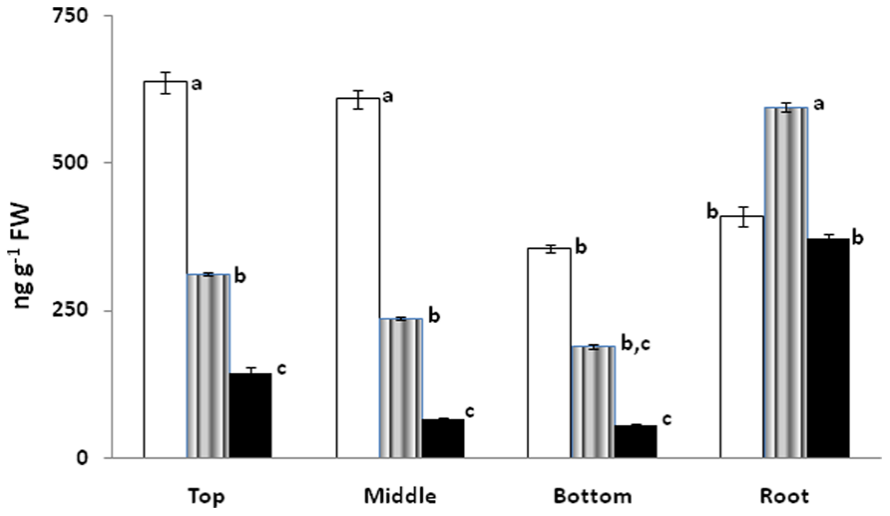
Total glucose content as observed in Kargil (□), Leh (∥∥) and Nyoma (▪) by the action of glucose oxidase. Means with the different letter represents statistically significant values at *p≤0.05* using ANOVA.

Total glucose content in the leaves of *Lepidium latifolium* differs significantly from location to location. Overall, the presence of higher amount of simple sugar like glucose makes it a good source of food. Leaves of the plants growing at Kargil showed higher amount of glucose content followed by plants in Leh (Fig. 4). Nyoma plants showed least amount of glucose in the leaves. The amount of glucose ranged between 55 to 637 ng g^−1^ fresh weight. Relatively, higher amount of glucose was found in roots as compared to leaves in plants growing in Leh and Nyoma. It appears that there is diversion of primary metabolites for the production of secondary metabolites. This is substantiated by relatively higher sinigrin content in Leh. Plants, in fact have limited resources to support their biochemical processes, hence, all requirements cannot be met simultaneously and trade-offs occur between growth and defence [38], [39]. We have earlier seen this trade-off between expression of antioxidant enzymes and secondary metabolites in *Swertia chirata* [40].

**Figure 5:**
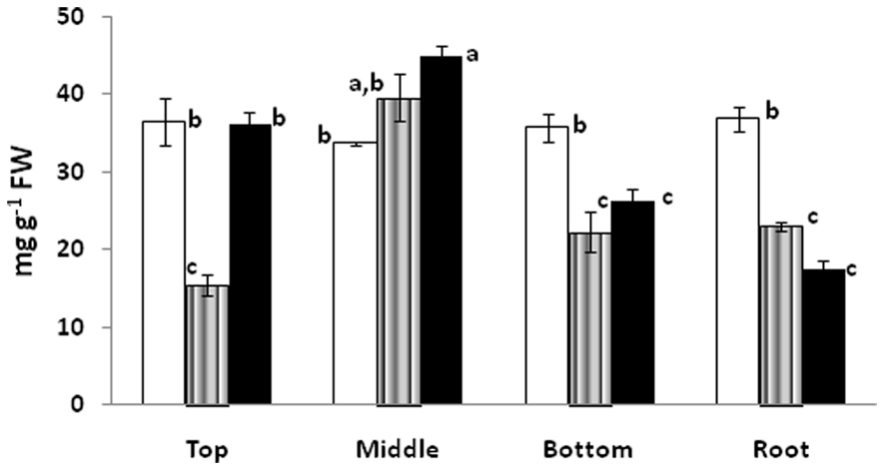
Total crude protein as observed in Kargil (□), Leh (∥∥) and Nyoma (▪) using Bradford reagent. Means with the different letter represents statistically significant values at *p≤0.05* using ANOVA.

Protein content on the other hand was found to be very high for this plant (Fig. 5). Percentage of total crude protein was found to be in the range of 1.74% to 4.49% of fresh weight. Higher content of protein would be an added advantage in any food product for want of amino acids. In one study on the nutritional evaluation of feeds and fodders of Ladakh, *Lepidium latifolium* was found to have highest crude protein on dry matter basis [41].

### Total phenol and ﬂavonoid content

Phenolic compounds are commonly found in plant kingdom that are associated with their redox properties, which allow them to act as reducing agents, hydrogen donors, and singlet oxygen quenchers [42]. As shown in table 4, the amount of total phenols ranged from 17.92 to 50.51 mg GAE g^−1^ dry weight in different sites, with maximum value found in Nyoma bottom leaves. Roots contain lesser phenolics than leaves. Similarly, the amount of ﬂavonoids varied significantly (*p≤0.05*) in different samples. Total flavonoids were found highest in Leh leaves with top leaves showing the highest value of 76.00 mg QE g^−^1 dry weight (Table 4). Roots of all places showed lesser amount than leaves. Flavonoids are widespread plant secondary metabolites, including ﬂavones, ﬂavanols, and condensed tannins. The free radical-scavenging activity of ﬂavonoids is dependent on the presence of free OH groups especially 3-OH. It has been reported that plant ﬂavonoids that show antioxidant activity *in vitro* also function as antioxidants *in vivo* [43], [44]. Since, this is the first report on *Lepidium latifolium*, further efforts would be made to determine active flavanoid components.

**Table 4:** Antioxidant capacity in *Lepidium latifolium* suggested by total phenols, total flavanoids, reducing and chelation power in different leaf positions and roots at three different locations of Ladakh.

		Kargil	Leh	Nyoma
	*Top Leaf*	30.56±1.04^a^	36.87±3.25*^a b^	29.21±0.23^b^
**Total Phenols**	*Middle Leaf*	33.33±3.05^a^	41.13±1.40*^a^	31.69±1.82^b^
(mg GAE g^−1^)	*Bottom Leaf*	26.89±0.20*^b^	33.74±4.87*^b^	50.51±1.17*^a^
	*Root*	19.64±2.79^c^	17.92±0.83^c^	24.74±3.26*^d^
	*Top Leaf*	44.66±3.05*^b^	76.00±8.03*^a^	58.00±5.06*^b^
**Total flavanoids**	*Middle Leaf*	54.00±6.03^a^	58.66±3.05^b^	71.33±6.36*^a^
(mg QE g^−1^)	*Bottom Leaf*	38.66±3.05*^b^	68.66±4.26*^a^	54.00±4.16*^b^
	*Root*	20.67±6.11^c^	22.00±1.15^c^	36.00±6.36*^c^
	*Top Leaf*	0.68±0.04*^a^	0.82±0.02^b^	0.82±0.03^b^
**Reducing Power**	*Middle Leaf*	0.68±0.02*^a^	0.83±0.01^b^	0.89±0.03^a^
(*A_700_*)	*Bottom Leaf*	0.59±0.03*^b^	0.97±0.04*^a^	0.83±0.02*^b^
	*Root*	0.48±0.05^c^	0.55±0.06*^c^	0.46±0.06^c^
	*Top Leaf*	41.71±2.57*^d^	13.71±2.15*^b^	32.85±2.46*^b^
**Chelation Power**	*Middle Leaf*	48.85±2.47*^c^	8.28±3.96*^c^	38.57±4.05*^b^
(% Inhibition)	*Bottom Leaf*	51.71±0.49*^b^	28.28±3.92*^a^	21.42±3.014*^c^
	*Root*	59.99±0.52^a^	16.85±3.45*^b^	56.28±0.84^a^

mg GAE g^−1^: milligram gallic acid equivalents per gram dry weight of sample; mg QE g^−1^: milligram quercetin equivalents per gram dry weight of sample; *A_700_*: absorbance at 700 nm. Each value is a mean of a triplicate analysis performed on different samples. * represents statistically significant values among rows whereas, different letters represents significant statistical variations at *p≤0.05* among columns.

### Reducing and chelation power

The presence of reductants in plant extracts causes the reduction of Fe^3+^/Ferric cyanide complex to ferrous form [45]. The order of reducing power was higher in leaves than in roots. Kargil showed significantly (*p≤0.05*) lower reducing power than Leh and Nyoma, which showed similar values (Table 4). The reducing capacity of a compound may serve as a significant indicator of its potential antioxidant activity [46] and showed similar results as observed for phenols and flavanoids. On the other hand, chelation power showed almost reverse trend with roots showing the higher activity. Among various sites, significant differences were found in the sequestration of Fe^2+^ ions with Kargil showing the highest values (Table 4). The metal chelating capacity is significant since it reduces the concentration of transition metal ions affecting lipid peroxidation and formation of other secondary free radicals [47].

### Radical scavenging activity

**Figure 6:**
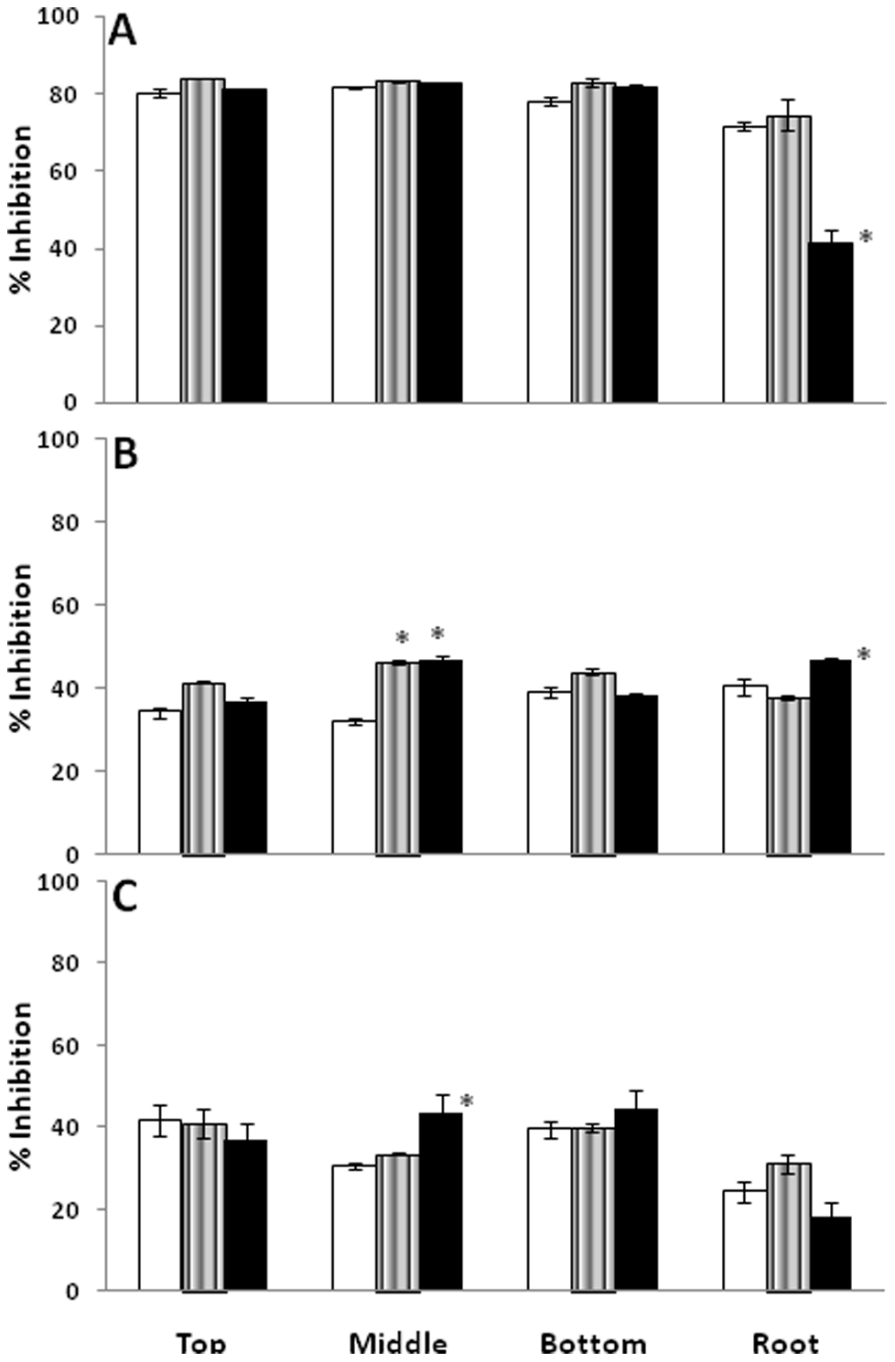
Antioxidant activity of methanolic plant extracts of *Lepidium latifolium* at Kargil (□), Leh (∥∥) and Nyoma (▪). Panel ‘A’ represents superoxide, panel ‘B’ represents hydroxyl and panel ‘C’ represents DPPH radicals. Antioxidant activity was represented as % inhibition of respective free radicals * represents statistically significant values at *p≤0.05*.

Three *in vitro* assays namely DPPH, O_2_^−^ scavenging activity and OH^·^ scavenging activity were performed to check the ability of plant extracts to quench free radicals. All the extracts have shown remarkable radical scavenging with their highest activity observed in superoxide radicals (Figure 6A). The percentage inhibition for O_2_^−^ scavenging activity ranged from 41.3% observed in Nyoma roots to 83.9% in Leh top leaves. Apart from singlet oxygen, superoxide radicals are the first products of oxidative chemistry that upon reaction, can further give rise to deleterious hydroxyl radical [4]. Efficient scavenging of O_2_^−^ radicals make this plant an effective antioxidant. The radical scavenging activity was almost similar in all leaf positions from different sites, with roots showing lesser activity. The values of OH^·^ and DPPH scavenging assay did not show significantly (*p≤0.05*) different values in the present study, which can be attributed to the fact that both are secondary radicals (Figure 6B, C).

### DNA protective activity

**Figure 7:**
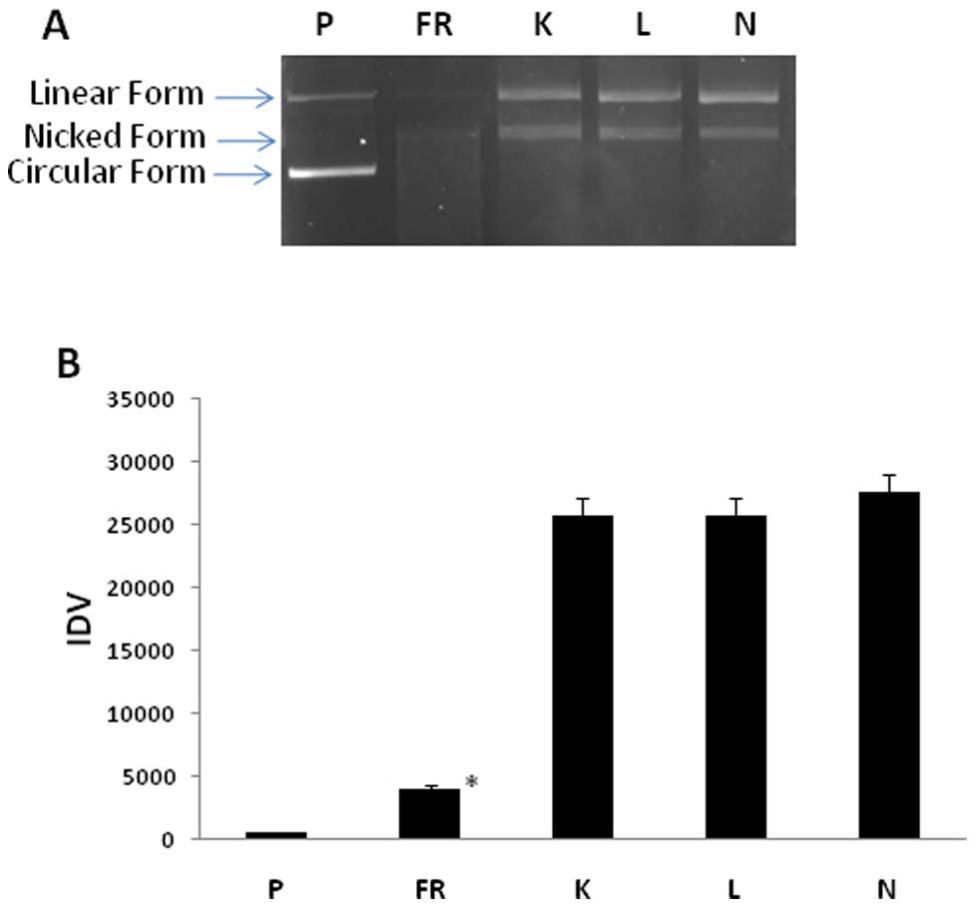
DNA protective activity of methanolic plant extracts of *Lepidium latifolium*. Panel ‘A’ represents 1.2% agarose gel stained with ethidium bromide visualised under UV whereas, panel ‘B’ represents Integrated Density Values (IDV) of bands using densitometry analysis. P: plasmid; FR: Fenton's reaction; K: Kargil; L: Leh; N: Nyoma.

Results on plasmid DNA showed significant amount of DNA protective activity among the top leaf extracts. As observed in figure 7, nicked and linear forms are clearly visible where extracts have been added, in comparison to the control samples where no extracts have been added. When DNA nicking activity was compared among the extracts from different sites, no significant difference was observed in their protective values. This is in accordance with the OH^·^ scavenging activity as earlier observed. Polyphenols with the ortho-dihydroxy structure in the B-ring (for example, catechin, epicatechin, and quercetin) have the highest scavenging activities [48] and their presence in this plant may contribute to the protective effect against free radical induced DNA damage.

## Conclusion

Our study showed that the ecotype of *Lepidium latifolium* present in Ladakh Himalayas does contain nutritionally significant levels of fatty acids and glucosinolates. The presence of high amount of sinigrin is of particular significance as its degradation product amounts to allyl isothiocyanates, which have known health alleviating properties. Leaves of this plant were found to contain among the highest amount of sinigrin among *phytofood*s. The plant was also reported to contain higher amount of linolenic acid and higher PUFA/SFA ratio, which is desirable for any food. The nutritive value of its leaves was found even higher than some of the widely used vegetable and fruits of family Brassicaceae. Apart from its nutritive properties, it was shown to contain substantial antioxidant activities. Although tender top leaves are mostly used in food preparations, our results showed that leaves at all the positions contain substantial nutritional and antioxidant properties (Table 5). Total amount of phenols and flavanoids were shown to be correlated with radical scavenging properties in this plant. It is therefore proposed that consumption of its leaves needs to be promoted as they can compensate to the dietary requirements of the local folk. This is of more significance given the fact that it is cold tolerant plant, leaves of which survive for the longest time during the harsh winters of Ladakh. This study is a first systematic evaluation of nutritive and antioxidant status of *Lepidium latifolium* from Ladakh.

**Table 5:** Position wise analysis of total protein, phenols, flavanoids and antioxidant capacity of leaves and roots in *Lepidium latifolium*.

	Top Leaf	Middle Leaf	Bottom Leaf	Root
***Total Protein***	29.3	39.4	28.1	25.8
***(mg g*** ^−***1***^ *** FW )***				
***Total Phenols***	32.2	35.4	37.0	20.8*
***(mg GAE g*** ^−***1***^ ***)***				
***Total Flavanoids***	59.6	61.3	53.8	26.2*
***(mg QE g*** ^−***1***^ ***)***				
***Antioxidant Capacity***				
***(% Inhibition)***				
**O_2_^·^** ^−^	81.8	82.6	80.8	62.4*
**OH^·^**	37.7	41.7	40.6	41.6
**DPPH**	39.9	35.9	41.4	24.6*

mg g-1 FW: mg protein (BSA) per gram fresh weight; mg GAE g^−1^: milligram gallic acid equivalents per gram dry weight of sample; mg QE g^−1^: milligram quercetin equivalents per gram dry weight of sample; Each value is a mean of all the values obtained for similar position at different locations. * represents statistically significant values among columns at *p≤0.05*
